# Oral fibropapillomatosis and epidermal hyperplasia of the lip in newborn lambs associated with bovine Deltapapillomavirus

**DOI:** 10.1038/s41598-018-31529-9

**Published:** 2018-09-06

**Authors:** Sante Roperto, Valeria Russo, Federica Corrado, Francesca De Falco, John S. Munday, Franco Roperto

**Affiliations:** 1 Dipartimento di Medicina Veterinaria e delle Produzioni Animali, Università di Napoli Federico II,via Delpino, 1 - 80137 Napoli, Italy; 2 Istituto Zooprofilattico Sperimentale del Mezzogiorno, via della Salute, 2 - 80055 Portici (Na), Italy; 3 Pathobiology, School of Veterinary Sciences, Massey University, Palmerston North, New Zealand; 4 Dipartimento di Biologia, Università di Napoli Federico II, 80126 Napoli, Italy

## Abstract

Congenital fibropapillomatosis of the gingiva and oral mucosa and epidermal hyperplasia of the lip are described, for the first time, in two newborn lambs. Expression of the E5 oncoprotein of bovine deltapapillomavirus types 2 (BPV-2) and -13 (BPV-13) was detected in both fibropapillomas and the hyperplastic epidermal cells suggesting the BPV infection was the cause of the proliferative lesions. No DNA sequences of BPV-1 and BPV-14 were detected. Both BPV-2 and BPV-13 DNA were also amplified from peripheral blood mononuclear cells (PBMCs) of the newborn lambs’ dams. The concordance between BPV genotypes detected in the blood of dam and the oral and skin pathological samples of their offspring suggests that a vertical hematogeneous transmission was most likely source of BPV infection. Immunoblotting revealed the presence of E5 dimers allowing the viral protein to be biologically active. E5 dimers bind and activate the platelet derived growth factor β receptor (PDGFβR), a major molecular mechanism contributing to disease. The detection of E5 protein within the proliferating cells therefore adds further evidence that the BPV infection was the cause of the proliferative lesions seen in these lambs. This is the first evidence of vertical transmission of BPVs in sheep resulting in a clinical disease.

## Introduction

Papillomaviruses (PVs) are small, non-enveloped, double-stranded DNA viruses that infect mucosal and cutaneous epithelia in most animal species, and in humans^1^. While there are currently over 200 types of human papillomaviruses (HPVs) recognized, only 24 bovine papillomaviruses (BPVs) have been fully characterized^2^.

Based on the degree of nucleotide sequence diversity of the L1 gene, BPVs are classified in five genera: Deltapapillomavirus (δPV) (BPV-1, -2, -13, -14), Epsilonpapillomavirus (εPV) (BPV-5, -8), Xipapillomavirus (χPV) (BPV-3, -4, -6, -9, -10, -11, -12, -15, -17, -20, -23, -24), Dyokappapapillomavirus (dyoκPV) (BPV-16, -18, -22), and Dyoxipapillomavirus (dyoχPV) (BPV-7). BPV-19 and BPV-21 are currently unclassified^2–4^.

The bovine δPVs have some unique biological properties such as inducing fibropapillomas in their respective hosts^5^ and being able to cause trans-species transmission^1^. The bovine δPVs have been associated with sarcoids in horses^6,7^, African lions^8^, and domestic cats^9^. Furthermore, bovine δPVs have been found in cutaneous lesions of buffaloes^10–12^, Cape mountain zebras, giraffes and sable antelopes^13,14^. BPV-1 and BPV-2 DNA has also been detected in a squamous cell carcinoma of head and neck in a Connemara mare^15^, and in a series of cutaneous spindle cell tumors in horses^16^. Bovine δPVs are also associated with bladder tumors in cattle and buffalo^17–20^. Very recently, BPV-2 DNA only has been detected from ovine cutaneous wart lesions^21^.

Horizontal route is thought to be the predominant mode of papillomaviral transmission. However, there is a growing interest in vertical PV transmission^22^ although the clinical relevance of vertical spread remains uncertain^23^. In humans, PV DNA has been detected in amniotic fluid, placenta and umbilical cord blood samples. This supports the possibility of in utero PV infection^24,25^ and vertical infection of PVs has been suggested to occur in up to 20% of people^25^. However, although PVs appear to be able to be transmitted vertically, such congenital infections rarely cause clinically visible lesions in children at birth^23^. In the non-human species, both abortive and productive infections of BPV-2 have been detected in the placenta of pregnant cows^26^. In addition, BPV-2- and BPV-13-associated placental papillomatosis was recently reported in buffaloes^27^.

In the present paper two newborn lambs that had congenital fibropapillomatosis of the gingiva and oral mucosa and epidermal hyperplasia of the lip are described. Both lesions were found to contain BPV-2 and BPV-13 DNA and E5 oncoprotein expression. To the authors’ knowledge, congenital PV-associated lesions have never been previously reported in sheep. Additionally, this is the first evidence that BPVs may be able to be transmitted vertically, and cause disease, in sheep.

## Results

### Clinical and gross pathology examination

Clinical examination of lamb No. 1 revealed a 4 × 5 cm area of roughened, reddish painful proliferative tissue, within the rostral mandibular gingiva, that appeared to have impeded the eruption of the central incisors and caused displacement of the remaining teeth. The opposing skin of the upper lip of this lamb was thickened and covered by thick scaling crusts (Supplemental Fig. S1). Clinical examination of lamb No. 2 revealed a 0.5 × 1 cm smooth and reddened proliferation within the gingival mucosa extending at both facial and lingual levels from the lower incisors. The gingival involvement and rapid growth of proliferative tissue resulted in displacement of the incisor teeth (Supplemental Fig. S2).

### Microscopic and ultrastructural findings

**Figure 1:**
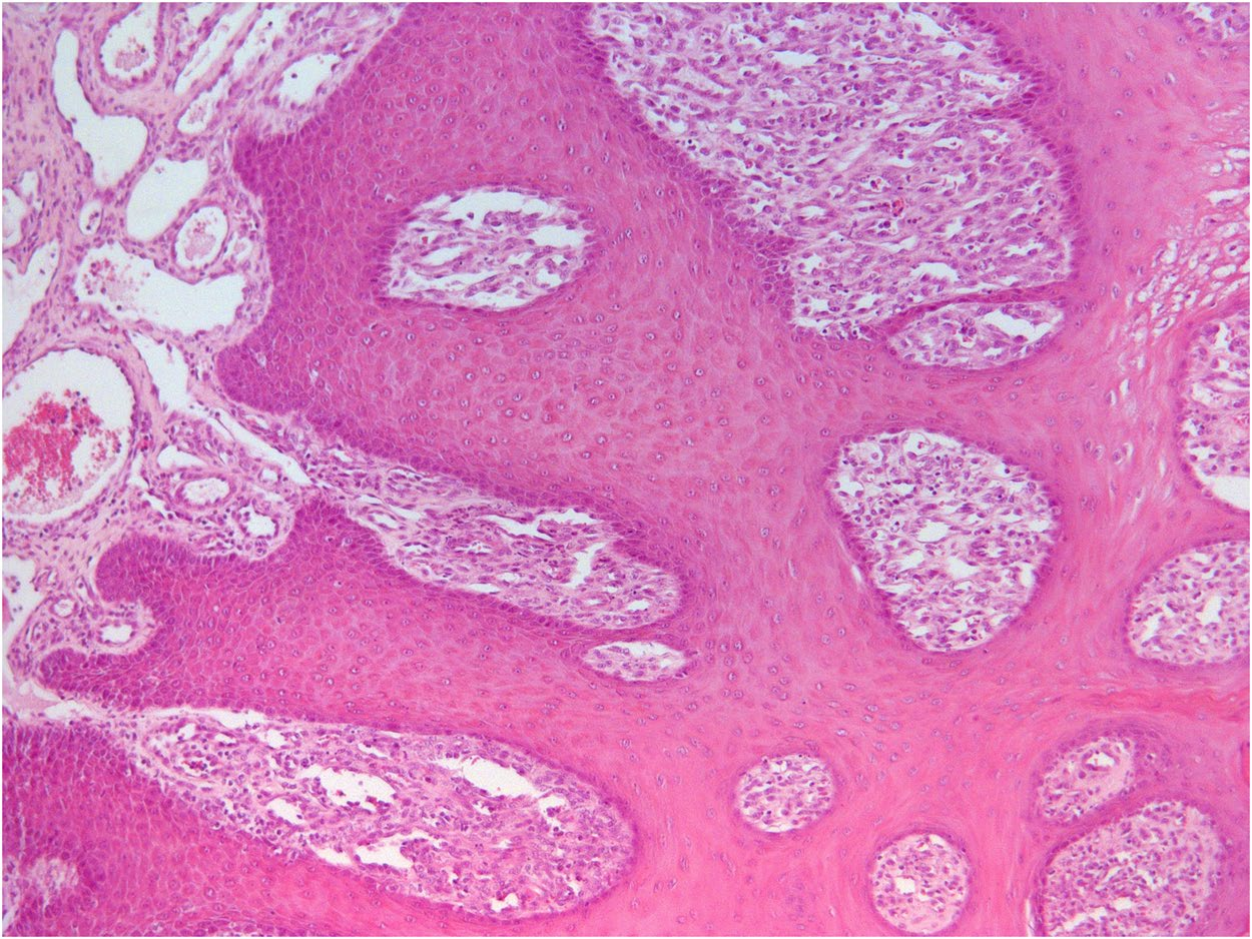
Oral fibropapillomatosis of lambs. Orderly proliferation of well-differentiated keratinocytes causing branched or fused rete ridges and supported by stalks of stromal fibroblasts is shown.

**Figure 2:**
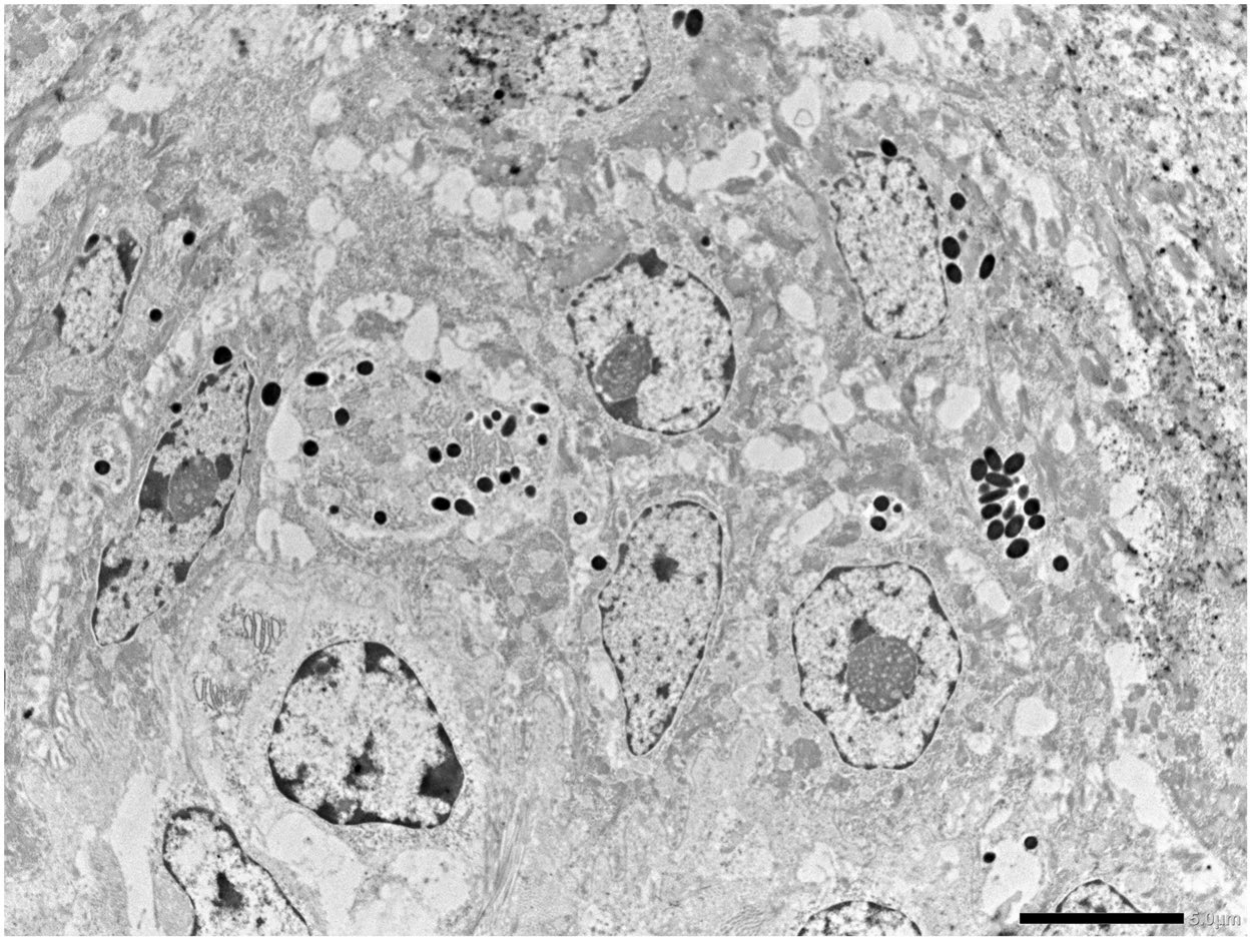
Oral fibropapillomatosis of lambs. Ultrastructural aspect of well differentiated keratinocytes containing phagocytic compound melanosomes. Notice marginated and central enlarged nucleolar fibrillar centers.

The proliferative tissue seen in the mandibular rostral gingiva of lamb No. 1 as well as the proliferation of gingiva and oral mucosa of lamb No. 2 was characterized by a papillary or acanthotic growth of well-differentiated squamous keratinocytes that retained an orderly maturation pattern. Epithelial hyperplasia was supported by marked stromal stalks composed of plump, haphazard fibroblasts with round vesicular nuclei and new vessel-forming endothelial cells. The hyperplastic epithelium extends as branches or rete pegs into the underlying submucosa (Fig. 1). Numerous mitotic figures were seen; some of them showed a spindle orientation that was perpendicular and/or oblique to the intact basement membrane (BM) resulting in asymmetric cell divisions (ACDs). Ultrastructurally, the proliferating cells were well-differentiated keratinocytes, often containing compound melanosomes. Most of the keratinocytes showed prominent nucleoli because of enlarged fibrillar centers (Fig. 2). Poorly developed desmosomes, known also as attenuated desmosomes, being the intermediate electron dense line lacking and causing the cells to lose their cohesiveness, were seen. Furthermore, some desmosomes appeared to have an abnormal size (giant desmosomes) (Supplemental Fig. S3). Intranuclear microtubular inclusions were detected in numerous keratinocytes. Asymmetric mitosis was documented by transmission electron microscope (TEM) showing the formation of two daughter cells of unequal size (Supplemental Fig. S4).

**Figure 3:**
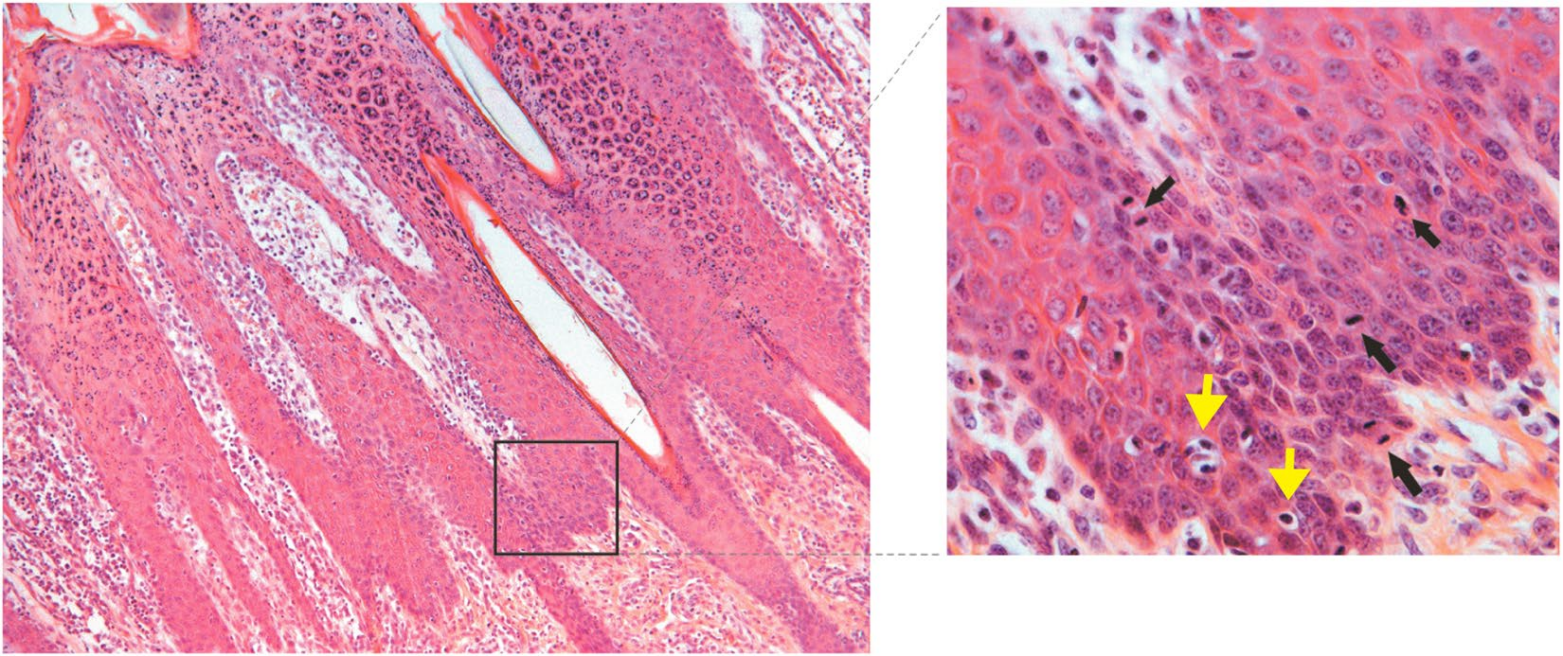
Epidermal hyperplasia of the lip of lambs. Epithelial projections representing expansion of the follicular infundibulum is shown. Several koilocytes (yellow arrows) and numerous symmetric and asymmetric mitoses are shown (black arrows) (inset).

Irregular epidermal hyperplasia covered by orthokeratotic hyperkeratosis was seen on examination of the skin from the lip. The epithelial projections commonly appeared to represent expansion of the follicular infundibulum. Numerous keratinocytes with shrunken nuclei surrounded by a clear cytoplasmic halo (koilocytes) and a high number of symmetric and asymmetric mitotic figures in basal and suprabasal layers were seen (Fig. 3). ACDs were clearly shown by the formation of micronuclei by TEM (Supplemental Fig. S5). Some attenuated as well as giant desmosomes and desmosomes-like structures were also seen in some keratinocytes of the stratum spinosum which sometimes appeared to have ring-shaped nucleoli. A diffuse cell hyperplasia of adnexal epithelium was also present. Inflammation characterized by neutrophils, eosinophils, lymphoid cells, and macrophages was seen in the dermis.

### Virological analysis

The presence of papillomatous hyperplasia within the lesions and clinical history of the flock suggested a possible role of viral agents in disease development. Hyperplastic lesions of the mouth and skin can develop in the disease contagious ecthyma (also known as scabby mouth and contagious pustular dermatitis) that is caused by the orf parapoxvirus, (OrfV). However, the presence of koilocytosis in the present cases was considered as indication of a papillomavirus infection. PCR screening revealed infection by bovine δPVs. PCR analysis failed to amplify DNA using primers for ovine OrfV in pathological tissue samples. Furthermore, neither DNA fragments from three ovine δPVs (OaPV-1, -2 and -4), known to be involved in cutaneous fibropapillomas of sheep^28^ nor OaPV-3, a dyoκPV associated to ovine squamous cell carcinoma^29^ were amplified. DNA was amplified as expected from positive control tissues using these primers.

**Figure 4:**
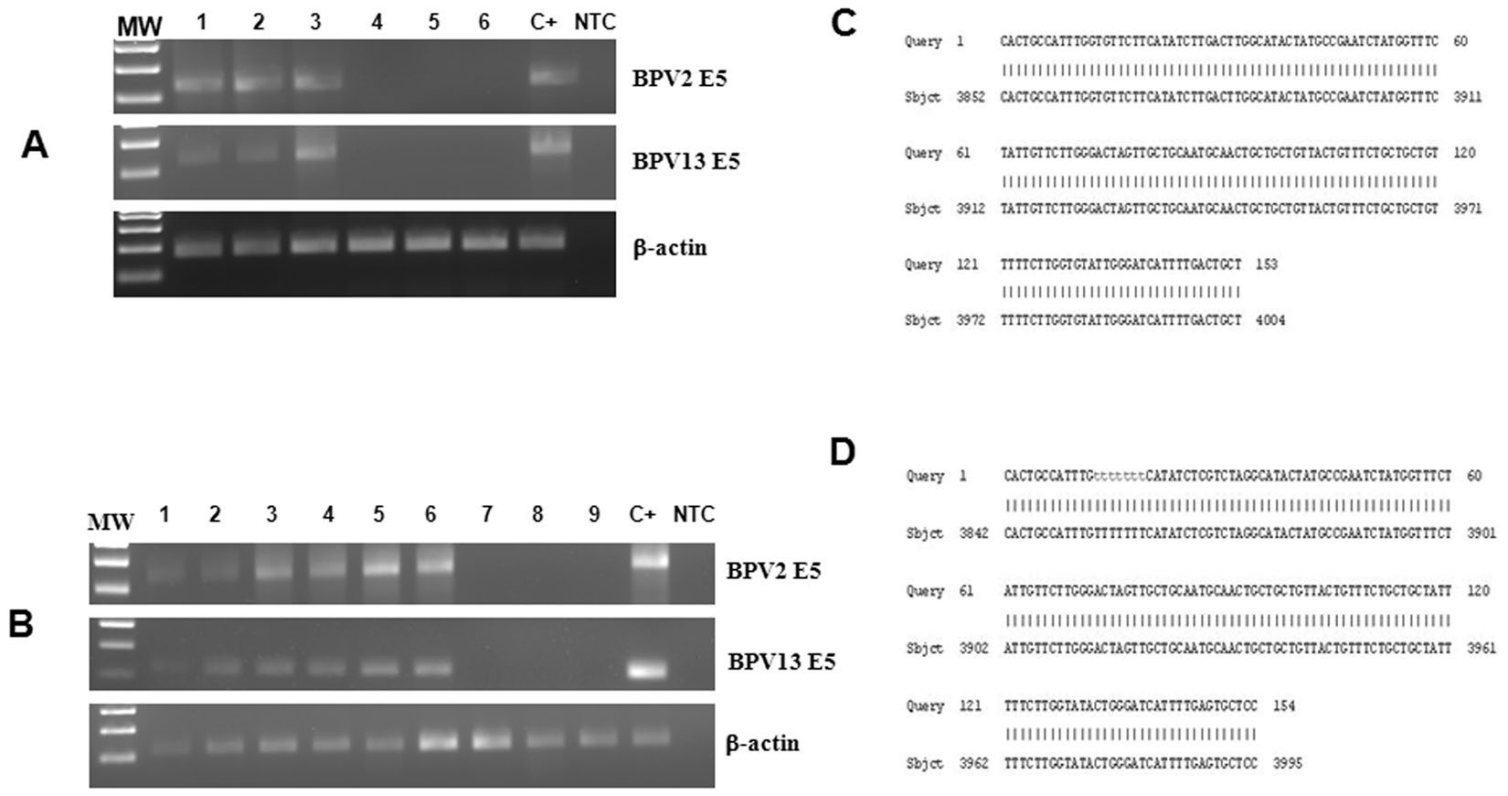
(**A**) PCR of DNA extracted from oral and skin lesions of newborn lambs. Lane 1 gingiva, Lane 2 palate, Lane 3 lip, lanes 4–6 healthy, non-infected lambs, Lane C+ positive control (BPV-2 and -13 E5-expressing urothelial cancer samples), Lane NTC: no template control. (**B**) RT-PCR: Lane 1 gingiva, Lane 2 palate, Lane 3 lip, Lane 4 spleen, Lane 5 kidney, Lane 6 liver, Lanes 7–9 healthy, non-infected lambs, Lane C+ positive control (BPV-2 and -13 E5-expressing urothelial cancer cells), Lane NTC- no template control. Sequencing results of both DNA and cDNA amplicons. Alignment of the sequences detects a 100% identity with E5 bovine papillomavirus type 2 (Sequence ID: M20219.1) in (**C**) and type 13 (Sequence ID: JQ798171.1) in (**D**).

As bovine δPV DNA has been found in peripheral blood mononuclear cells (PBMCs) of the dams of these lambs^30^, we addressed to detect the expression of these viruses, which may have had a potential role in causing this pathology. Neither BPV-1 nor BPV-14 E5 DNA was amplified from any tissue samples. However, fragments of BPV-2 and BPV-13 E5 DNA were amplified from fibropapillomas and hyperplastic skin samples. Sequencing of the amplicons unveiled DNA 154 bp and 153 bp sequences that showed a 100% identity with the known sequences of BPV-2 E5 DNA deposited in GenBank (MF045490.1) and of BPV-13 E5 DNA (GenBank: KU519390.1), respectively (Fig. 4).

### Reverse transcription (RT)-PCR

To evaluate whether or not BPV-2 and BPV-13 were transcriptionally active, the possible presence of transcripts of E5 oncoprotein, the major oncoprotein of the bovine δPVs, was investigated. RT-PCR showed both BPV-2 and BPV-13 E5 transcripts in fibropapilloma and hyperplastic skin samples from both lambs. BPV-2 and BPV13 E5 transcripts were also seen in liver, kidney and spleen samples. The amplified RNA was sequenced and had a 100% identity with BPV-2 and BPV-13 sequences (Fig. 4).

### Western blot and immunoprecipitation

Immunoblotting performed on samples of liver, spleen and kidney revealed the presence of E5 dimers and monomers (Supplemental Fig S6). These results were also confirmed by immunoprecipitation studies.

### Immunohistochemistry

To evaluate the morphological expression of E5 oncoprotein, an immunohistochemical study was performed both in oral and skin lesions. E5 proteins are the most highly conserved proteins encoded by δPVs^31^.

**Figure 5:**
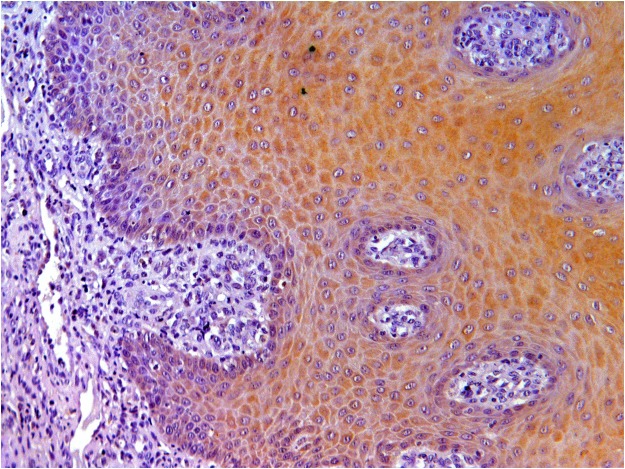
Oral fibropapillomatosis of lambs. A strong E5 immunolabeling is shown in the cytoplasm of epithelial cells only. No. E5 immunolabeling was seen in mesenchymal cells.

In the proliferative tissue samples from oral mucosa composed of epithelial and mesenchymal cells, E5 oncoprotein immunolabeling was only visible in the epithelial cells. The immunolabeling was prevalently cytoplasmic and membranous, although rare nuclear immunolabeling was also detected (Fig. 5 and Supplemental Fig. S7).

**Figure 6:**
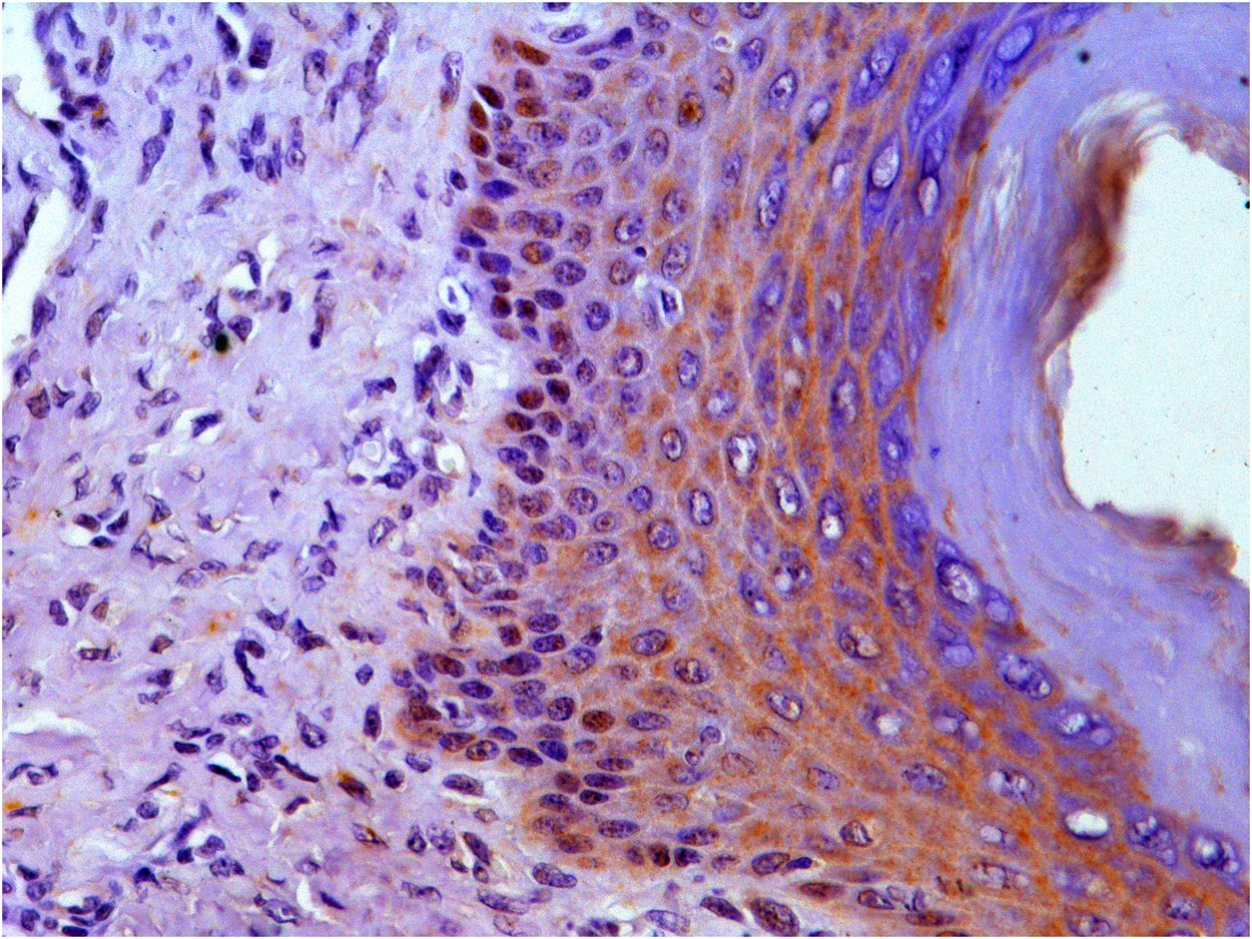
An immunolabeling for E5 oncoprotein is seen in the nuclei of the stratum basale; E5 immunolabeling is mainly seen in the cytoplasm of the epidermal cells of the stratum spinosum and granulosum.

E5 immunolabeling was also present in the hyperplastic epidermal cells and cells of skin adnexa. Immunolabeling was seen in nuclei of keratinocytes of the stratum basale, but in the cytoplasm of keratinocytes of the strata spinosum and granulosum (Fig. 6). Immunolabeling for E5 was present in cells of adnexal glandular epithelium; however, the most intense immunolabeling was in the cytoplasm of the hyperplastic cells of outer root sheath especially at isthmus level (Supplemental Fig. S8).

## Discussion

Microscopic patterns of the proliferative lesions of the gingival, oral mucosa, and the skin of the lip suggested diagnoses of oral fibropapillomatosis and epidermal hyperplasia, respectively. Both fibropapilloma and hyperplastic epidermal cells contained E5 DNA and RNA and the presence of oncoprotein of both BPV-2 and BPV-13 was detected in these lesions. These results suggest that one, or both, BPVs could have caused the proliferative lesions in these lambs. Indeed, immunoblotting studies detected a dimeric conformation of the viral E5 protein in some organs of these lambs. This suggests that E5 protein was present as a dimer which is known to be the biologically active conformation of the protein^32^. To the authors’ knowledge, BPVs have not been previously reported as a cause of clinical disease in sheep.

As the lambs were neonatal when they showed these lesions, BPV infection appears most likely to have been transmitted vertically. The concordance between BPV genotypes detected in the blood of the dams and the oral and skin pathological samples of their offspring supports a vertical hematogenous route of transmission rather than an ascending infection from the ewe or an infection at the time of parturition. Therefore, ovine placenta may play an important role in the transmission of BPVs, just several other viruses do in sheep. Vertical transmission of HPVs is well recognized and it has been found that the presence of HPV in placenta and/or in cord blood increased the risk of the newborn to carry HPV DNA in the oral mucosa^24,33,34^. To the authors’ knowledge, vertical transmission of a BPV in a sheep has never been previously reported.

This study shows, for the first time, that BPV-2 and BPV-13 could be involved in sheep pathology, thus representing a novel trans-species infection.

Congenital tumors occur sporadically in farm animals^35^ and fibropapillomatosis/papillomas have been reported at birth in cattle^36^, horses^37^, and pigs^38^. A viral origin for these congenital forms has been proposed but it has not been proven^37,39^, and these lesions are widely regarded to be more likely hamartomatous rather than infectious^37,38^. While further investigation is required, evidence from the currently reported lambs suggests that PVs can be transmitted vertically and therefore could cause more congenital tumors of farm animals than is currently recognized.

Ultrastructural and immunohistochemical findings from these lambs suggest a potential connection between BPV-associated carcinogenesis and the stem cell mode of division. There is some evidence that loss of regulation of ACD, a property of stem cells^40^, could be a key oncogenic event^41,42^. Indeed, it has been shown that a shift of the physiological asymmetric mode of division toward a symmetric one appears to occur in oral and mammary stem cells resulting in cancer stem cell (CSC) formation^42–44^.

In the present report, ACD was detected in both oral and cutaneous lesions. To our knowledge, no previous reports of PV-associated ACD have been documented in domestic mammals. Very rarely, mitotic asymmetries have been reported in laboratory mammals and it is unknown whether this mechanism has been well conserved^45^. Furthermore, a strong BPV E5 immunoreactivity was detected in the nuclei of interfollicular keratinocytes of stratum basale, some cells of which have known to have property of stem cells^46–48^ and in the cytoplasm of cells of outer root sheath (ORS) of hair follicle. The latter is an area particularly rich in self-renewal stem cells since ORS cells of sheep originate from bulge-derived keratinocytes, known to be hair follicle stem cells (HFSCs)^49^. It is tempting to speculate that, such as HPVs^50–52^ and rabbit oral papillomavirus (ROPV)^46^, the reservoir for BPV latent infection may reside within keratinocytes that have stem cell properties. Therefore, BPVs may interfere with ACD homeostasis generating a pool of stem cells that may be predisposed to cell transformation. Such connections between ACD and PV tumorigenesis have begun to emerge from *in vitro* and *in vivo* models^53–56^ although future studies are needed to gain insights into the relationship, if any, between PV-associated tumorigenesis and ACD perturbation.

In conclusion, this is the first time that PV-associated congenital lesions have been observed involving the oral cavity and skin of sheep. The oral lesions were so severe that they resulted in death of both lambs. The ability of BPVs to cause disease in sheep has significant implications in disease prevention when sheep are co-grazed with cattle.

## Methods

### Ethics statement

In this study we did not perform any animal experiments.

### Animals

The owner of a sheep flock composed of two hundred dairy Sardinian sheep reported that five lambs, singletons from five ewes, had unusual oral lesions characterized by proliferative tissues in the gingiva and oral mucosa that were present at birth. Two of the five lambs were examined at the Istituto Zooprofilattico Sperimentale of Sassari when ten- and twelve-day-old respectively. As the lesions appeared to interfere with suckling, both lambs were extremely emaciated and died despite attempts at treatment within a few days. Both lambs were necropsied. One of the lambs also had thickened skin of the upper lip that was covered by a thin serocellular crust. No other significant lesions were observed on necropsy examination of the lambs. The flock had close contact with cattle and grazed on lands contaminated with bracken fern. Cattle from the same pastures that the sheep had grazed had previously been reported to develop chronic enzootic hematuria and BPV-associated bladder tumors. Furthermore, oral mucosa and lip samples from healthy, non-infected lambs were obtained at public slaughterhouses.

### Light microscopy

Oral proliferative tissue and lip skin samples were fixed in 10% neutral buffered formalin and routinely processed for histological examination. Microscopic examination was assessed on 5-µm-thick hematoxylin-eosin (HE)-stained sections. The sections were observed with a Leica DM 4000 B optical microscope equipped with a Leica DMC4500 digital microscope camera (Leica Microsystems CMS GmbH-DCC, Wetzlar, Germany).

### Transmission electron microscopy (TEM)

Oral proliferative tissue and lip skin samples were immediately fixed in 4% glutaraldehyde in 0.1 M phosphate buffer (pH 7.4) for 2–3 h. They were washed (20 min 5 times) and post fixed in 1% OsO4 in the same buffer for 1 h. They were washed again in 0.1 M phosphate buffer (pH 7.4) and then dehydrated in graded alcohol and embedded in Agar Low Viscosity Resin (Agar Scientific Limited, Essex, England). Semi-thin section (400 nm) were cut on an EM UC6 ultramicrotome (Leica Microsystems) and were stained with 1% toluidine blue in water solution and examined by Leica DM 4000 B optical microscope. Ultra thin sections (60–70 nm), obtained from chosen areas, were collected onto 300-mesh grids coated with formvar and counterstained with lead citrate and uranyl acetate. The sections were observed with a JEOL JEM-1011 transmission electron microscope (JEOL, Tokyo, Japan) equipped with a thermionic tungsten filament and operated at an acceleration voltage of 100 kV. Images were taken using a Morada cooled slow-scan CCD camera (3783 × 2672 pixels) and micrographs were taken with iTEM software (Olympus Soft Imaging System GmbH, Munster, Germany).

### DNA extraction and PCR amplification

Total DNA was extracted from gingiva and oral mucosa and lip skin samples from both lambs and from three healthy, non-infected lambs using a DNeasy Blood & Tissue Kit (Qiagen TM, ME, DE), according to the manufacturer’s instructions. PCR was performed with 100 ng of DNA. Specific primer sets for three ovine and four bovine δPVs, for ovine parapoxvirus OrfV and for Ovis aries papillomavirus 3 (OaPV3), a novel ovine dyoκPV, were used (Supplemental Table S1). Conditions for PCR were: 94 °C for 10 min, 40 cycles of 95 °C for 30 s, 56 °C for 30 s and 72 °C for 30 s. The positive control sheep tissues for OrfV were from molecular laboratory of the National Reference Center for Whole Genome Sequencing of Microbial Pathogens at the Istituto Zooprofilattico Sperimentale di Teramo (a kind gift from Dr Alessio Lorusso). The positive control tissues for ovine δPVs and dyoκPV were ovine cutaneous fibropapillomatosis and ocular squamous cell carcinoma samples, respectively, from Department of Veterinary Medicine of Sassari University (a kind gift from prof. A. Alberti). The positive control tissues for bovine δPVs were urothelial tumor samples of the urinary bladder of large ruminants from the Department of Veterinary Medicine, Naples University^18,19^. For each sample, the PCR experiment was repeated in triplicate to validate the accuracy of the obtained data.

### Reverse Transcription (RT)-PCR

Total RNA from gingiva and oral mucosa and lip skin was extracted from formalin-fixed paraffin embedded (FFPE) tissues (five sections about 20 μm thick) using a Qiagen FFPE RNeasy Kit (Qiagen Cat #74404, Valencia, CA, USA). After deparaffinization, the remaining steps of RNA extraction were followed according to the manufacturer’s instructions. Total RNA was also extracted from frozen liver, kidney and spleen samples by RNeasy Mini Kit (Qiagen TM, 74104), in according to the manufacturer’s instructions. All RNA samples, including oral mucosa and lip samples from healthy, non-infected lambs, were analyzed spectrophotometrically on a Nanodrop (NanoDrop 1000 Spectrophotometer V3.7). Five hundred nanograms of the total RNA was used to generate the first strand of cDNA by the QuantiTect Reverse Transcription Kit (205311, Qiagen) in to according the manufacturer’s instructions. PCR investigations were performed as above described. For each sample, RT-PCR was repeated in triplicate to validate the accuracy of the obtained data.

### Sequence analysis

PCR products from DNA and cDNA, were purified using a Qiaquick PCR purification Kit (Qiagen TM, ME, DE) and bidirectionally sequenced using a BigDye_Terminator v1.1 Cycle Sequencing Kit (Applied Biosystems, CA, USA) following manufacturer’s recommendations. Sequences were dye-terminator removed by DyeEx_ 2.0 spin kit (Qiagen TM, ME, DE) and run on a 3500 Genetic Analyzer (Applied Biosystems, CA, USA). Electropherograms were analyzed using Sequencing analysis v5.2 and sequence scanner v1.0 softwares (Applied Biosystems, CA, USA). The sequences obtained were compared to others in GenBank using the BLAST program.

### Western blot and immunoprecipitation

Western blot and immunoprecipitation analyses were only performed on liver, kidney and spleen samples from two lambs. The positive control tissues for bovine δPVs were from urothelial tumors of the bovine and bubaline urinary bladder from the Department of Veterinary Medicine, Naples University^18,19^. They were lysed in immunoprecipitation assay-morpholinepropanesulfonic acid (RIPA-MOPS) buffer (20 mM MOPS, 150 mM NaCl, 1 mM EDTA, 1% NP-40, 1% deoxycholate, and 0.1% SDS) containing protease and phosphatase inhibitors and extracted proteins were quantified by Bradford assay. For E5 immunoprecipitation, 10 μl of an anti-E5 rabbit polyclonal antiserum recognizing the C-terminal 14 amino acids of the BPV E5 protein (a kind gift provided by Prof. DiMaio, Yale University, New Haven, USA) was added to 1 mg of protein. Protein-antibody mixtures were overnight at 4 °C, and then incubated with protein A-Sepharose beads (GE Healthcare) for 1–2 h at 4 °C. The beads were then pelleted, washed, and resuspended in 2× Laemmli sample buffer. Immunoprecipitates were boiled to elute immune complexes and electrophoresed for 1.5 h at 150 V on a 15% (wt/vol) polyacrylamide/SDS gel. Samples were transferred for 1.5 h at 100 V to PVDF membranes in transfer buffer (25 mM Tris base, 192 mM glycine, and 20% (vol/vol) methanol). Membranes were blocked in 5% (wt/vol) nonfat dry milk in TBST (10 mM Tris ⋅ HCl (pH 7.4), 167 mM NaCl, 1% Tween-20) for 1 h and incubated overnight at 4 °C with anti-E5 antibody diluted 1:1,250, in 5% (wt/vol) milk/TBST. Blots were washed five times in TBST and subsequently incubated for 1 h at room temperature with horseradish peroxidase (HRP)-conjugated donkey anti-rabbit secondary antibody diluted 1:3000, in 5% (wt/vol) milk/TBST. Blots were then washed and visualized by enhanced chemiluminescence.

### Immunohistochemistry

Immunohistochemical investigation (IHC) was carried out on both pathological oral and skin samples using an anti-E5 rabbit polyclonal antiserum recognizing the C-terminal 14 amino acids of the BPV E5 protein (a kind gift by Prof. DiMaio, Yale University, New Haven, USA). Wax sections, 5-µm-thick, were prepared from each waxed tissue sample. The sections were deparaffinized in xylene and rehydrated in graded ethanol. Antigen retrieval was performed by pretreating with microwave heating (twice for 5 min each at 700 W) in citrate buffer pH 6.0. All the slides were allowed to cool at room temperature and washed gently three times with phosphate buffered saline (PBS, pH 7.4, 0.01 M), then incubated for 20 min at room temperature with Peroxidase Blocking solution (Vector Laboratories Inc., CA, USA). Then, the sections were incubated for 1 h with normal goat serum (Vector Laboratories Inc., CA, USA) diluted at 20% in PBS.

After tapping off the excess serum, the anti-E5 rabbit polyclonal antiserum diluted at 1 in 300 in PBS, was applied for 1 h and 30 min at room temperature in a humid chamber. The slides were washed gently three times with PBS, then incubated for 30 min with appropriate biotinylated secondary anti-rabbit antibody (Vector Laboratories Inc., CA, USA) diluted at 1 in 200 in PBS. Sections were washed three times with PBS and then incubated with Vectastain ABC reagent (Vector Laboratories Inc., CA, USA) in a humid chamber at room temperature for 30 min. Color development was obtained by treatment with 3,3′-diaminobenzidine (DAB) (Vector Laboratories Inc., CA, USA) for 2–10 min. Sections were counterstained with Mayer’s hematoxylin. The sections were observed with a Leica DM 4000 B optical microscope equipped with a Leica DMC4500 digital microscope camera (Leica Microsystems CMS GmbH-DCC, Wetzlar, Germany).

The anti-E5 rabbit polyclonal antiserum specificity was shown by using sections from the same pathological tissue samples as negative control where the antiserum was omitted and replaced by a normal rabbit serum (Vector Laboratories Inc., CA, USA). Further positive control sections were from urothelial neoplastic samples in which E5 oncoprotein is known to be expressed^18,19^.

## Electronic supplementary material

Supplementary information

## Data Availability

All data generated or analyzed during this study are included in this published article (and its Supplementary Information files).
