# Supplementary material for: Oral fibropapillomatosis and epidermal hyperplasia of the lip in newborn lambs associated with bovine Deltapapillomavirus
Source: Sci Rep. 2018 Sep 6;8:13310. doi: 10.1038/s41598-018-31529-9 (PMC6127103; doi:10.1038/s41598-018-31529-9)
Supplement: Supplementary file 1 — Supplementary information [file 41598_2018_31529_MOESM1_ESM.pdf]

Supplemental Table S1 - Table reports all primer sets used in this present study

Bovine Deltapapillomavirus	Sequence	bp	Amplicon size - bp	GenBank Acc.number
L1 BPV1 F	GGCTGAGGACGCTGCTGGTA	20	92	KX907623.1
L1 BPV1 R	TCTCCGAGCCCCCTCTGGTC	20		
E5 BPV2 F	CACTGCCATTTGTTTTTTTC	20	154	MF045490.1
E5 BPV2 R	GGAGCAACTCAAAATGATCCC	20		
E5 BPV13 F	CACTGCCATTTGGTGTTCTT	19	153	KU519390.1
E5 BPV13 R	AGCAGTCAAAATGATCCCAA	20		
L1 BPV14 F	GGAACAAACCTCACAATCAC	20	195	KR868228.1
L1 BPV14 R	CCAGTTCTCTAATACTGAGG	20		

OrfV

045F	CCTACTTCTCGGAGTTCAGC	20	392	KP010354.1
045R	GCAGCACTTCTCCTCGTAG	19		
PPP1	GTCGTCCACGATGAGCAGCT	20	594	KU884329.1
PPP4	TACGTGGGAAGCGCCTCGCT	20		
VIR1	ACAATGGCCTGCGAGTG	17	617	AJ222702.1
VIR2	TTAGAACTGATGCCGCAG	18		
PPV F	CATCCCCAAGGAGACCAACGAG	22	627	KP010355.1
PPV R	TCCTCGTCGCCGTCGAAGTC	20		

Ovine Papillomavirus

OaPV1 F	ACCGTTATCTAGAATCGCCAGC	23	194	U83594.1
OaPV1 R	CTCGCCTGCGCACTATTGTT	20		
OaPV2 F	ACCGACACCTGTGACCAAAA	20	174	U83595.1
OaPV2 R	ACTGGTTAGGATCGGGGAGT	20		
OaPV3, L1 F	AACTGGACTTGTCTTCCATG	20	126	FJ796965
OaPV3, L1 R	AAAGACTCGGTATTGGGAGG	20		
OaPV3, E6 F	AAGCCCTCGTACAATAGCTG	20	150	FJ796965
OaPV3, E6 R	GCCAAATCTCCAGAGTAAAGC	20		
OaPV4 F	CCAAGATGCTGAGCAGTAAATTCC	24	377	KX954121.1
OaPV4 R	TTATGGCTATTTGGTCCGTGC	21		

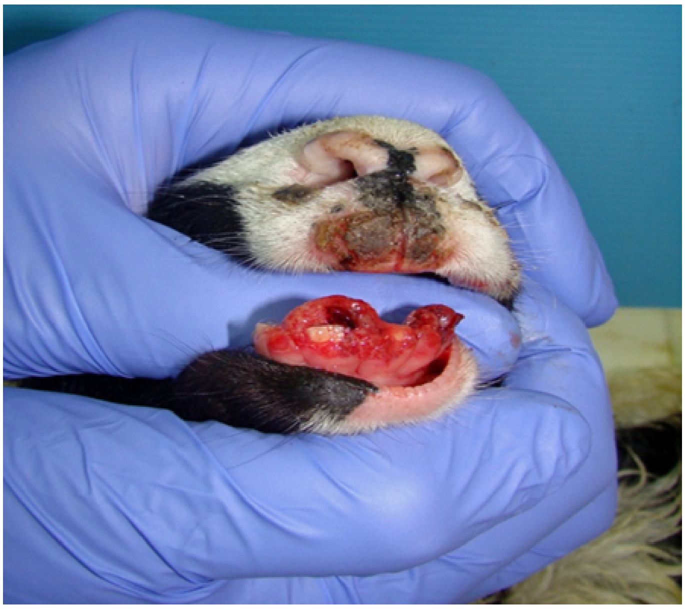

Suppl. Figure S1.

Clinical findings of epidermal hyperplasia of the lip and oral fibropapillomatosis of rostral mandibular gingiva of a newborn lamb.

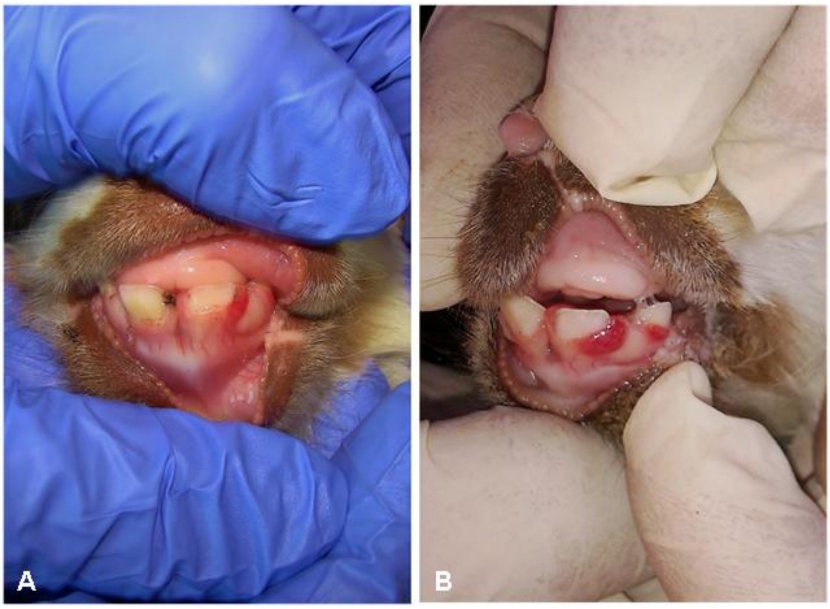

Suppl. Figure S2.

Gingival fibropapillomatosis in a lamb at 1 day of age (A) and 12 days later (B).

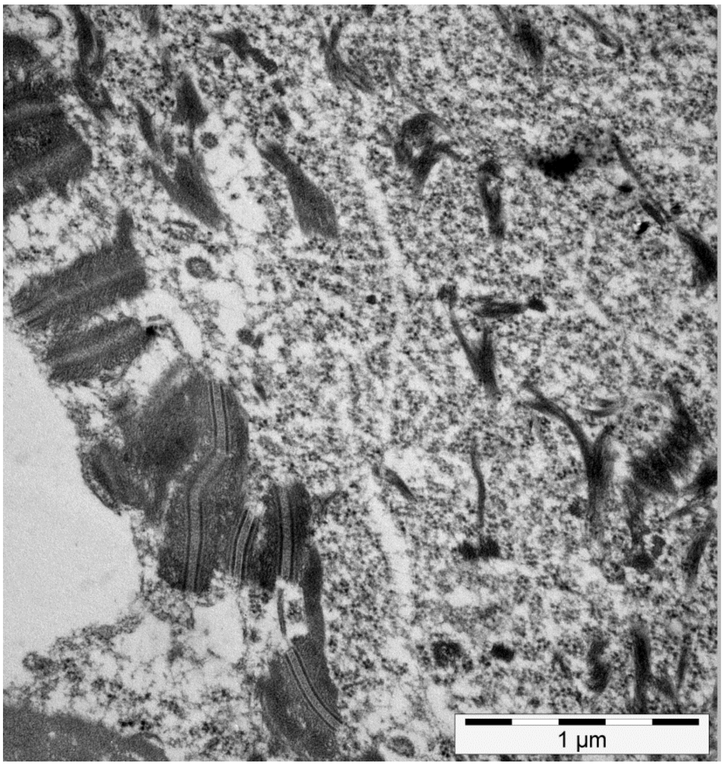

Suppl. Figure S3.

Several poorly developed desmosomes characterized by the lacking of the intermediate line are shown. Notice the presence of giant desmosomes.

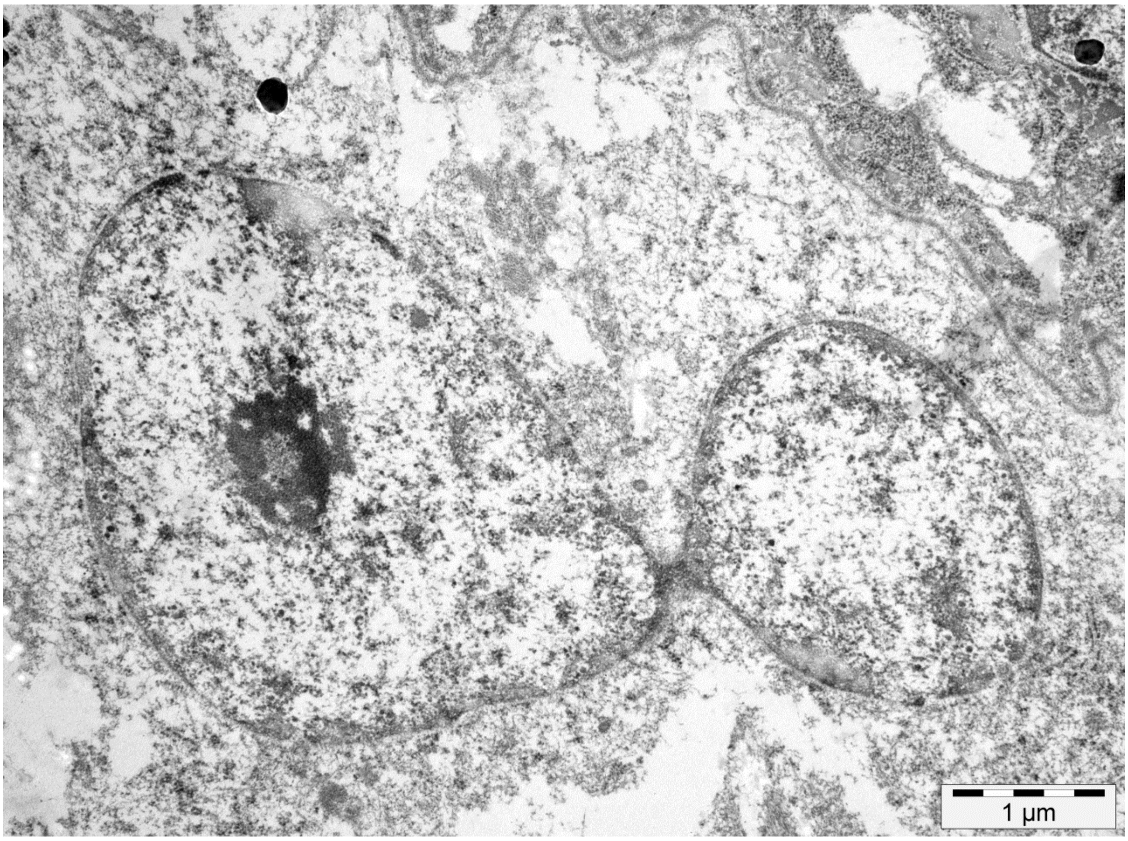

Suppl. Figure S4.

Oral fibropapillomatosis. An asymmetrycal cell division giving rise to two daughter cells of unequal size is shown by TEM.

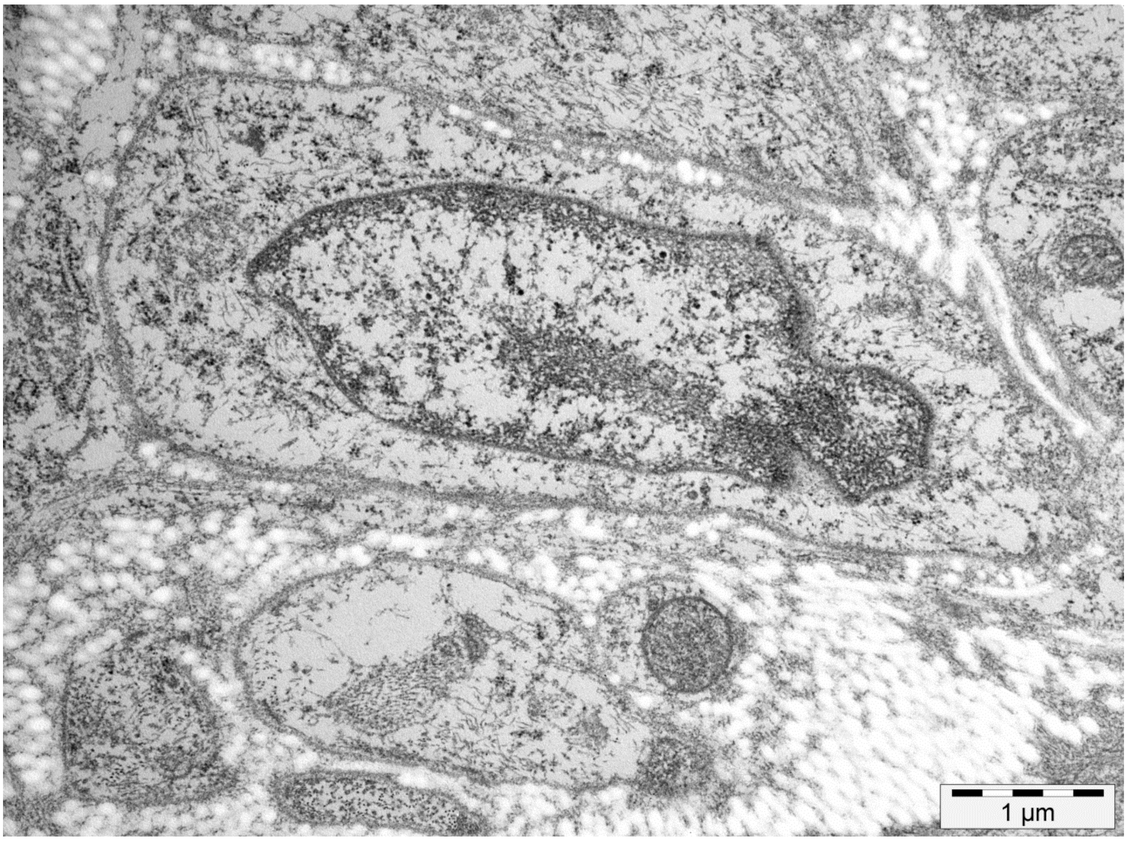

Suppl. Figure S5.

Epidermal hyperplasia. Ultrastructural detection of an asymmetric cell division resulting in the formation of micronuclei in the skin cells.

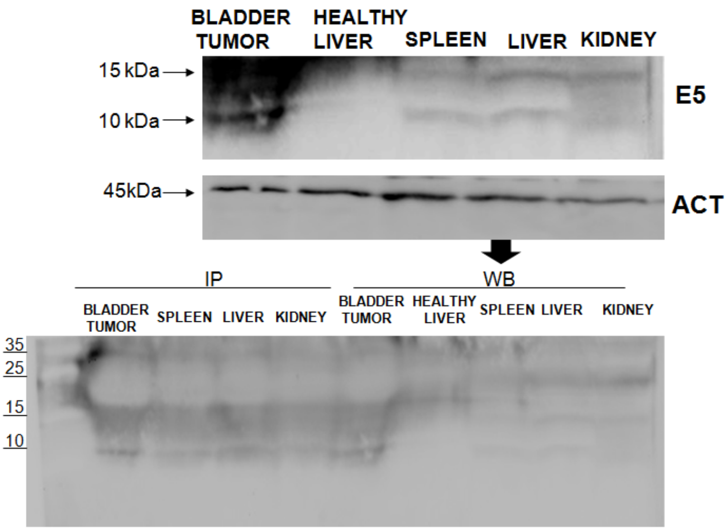

Suppl. Figure S6.

Immunoblotting of E5 oncoprotein in spleen, liver and kidney. Both E5 monomers (7kDa) and dimers (14 kDa) were evident in total extracts. Urothelial cancer cells expressing BPV-2 papillomavirus E5, were used as positive control; healthy liver tissue was used as negative control. Actin protein levels were detected to ensure equal protein loading.

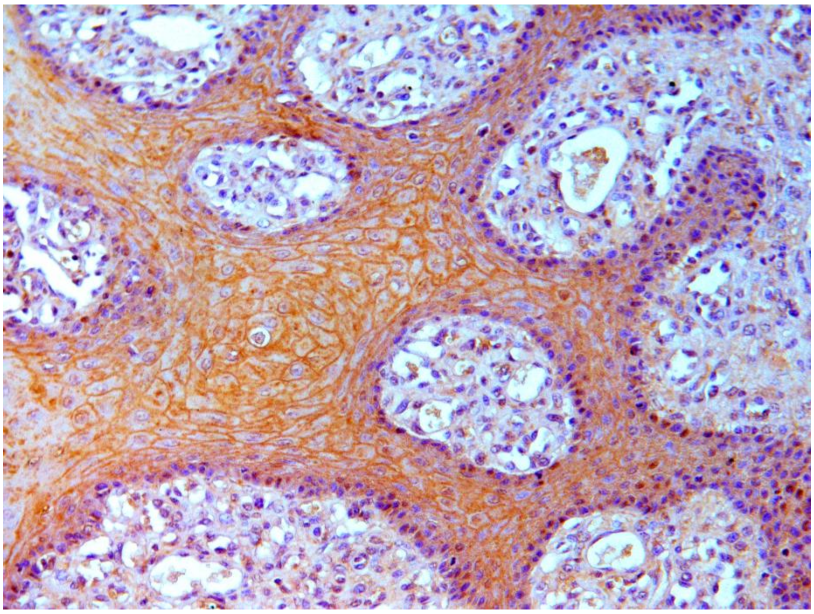

Suppl. Figure S7.

Oral fibropapillomatosis. A membranous pattern of E5 immunolabeling is clearly evident.

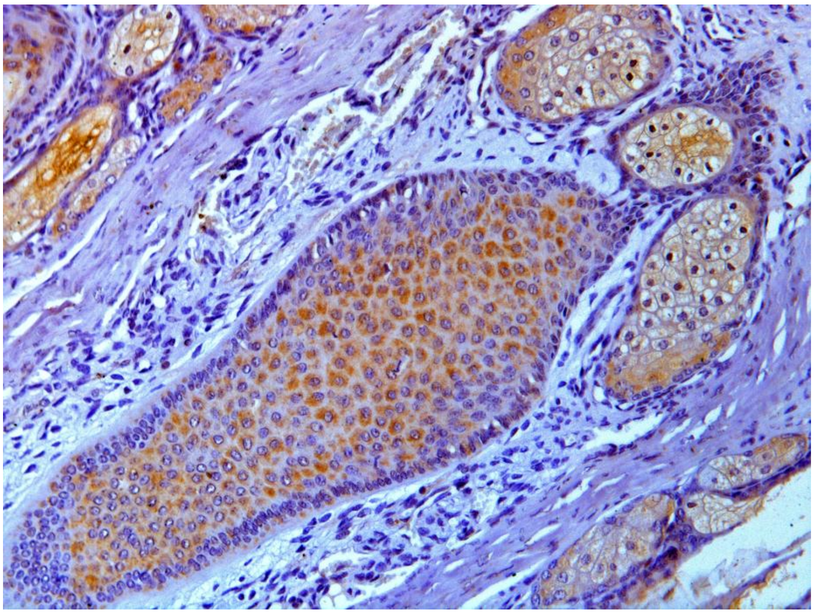

Suppl. Figure S8.

Epidermal hyperplasia. A strong E5 immunolabeling in acanthotic cells of the outer root sheath (OCS). Notice also E5 immunolabeling in the cells of sebaceous glands.
